# Supplementary material for: A b map implying the first eastern rupture of the Nankai Trough earthquakes
Source: Nat Commun. 2018 Mar 16;9:1117. doi: 10.1038/s41467-018-03514-3 (PMC5856758; doi:10.1038/s41467-018-03514-3)
Supplement: Supplementary file 1 — Supplementary Information(PDF 6802 kb) [file 41467_2018_3514_MOESM1_ESM.pdf]

Supplementary Information

A *b* map implying the first eastern rupture of the Nankai Trough earthquakes

K. Z. Nanjo & A. Yoshida

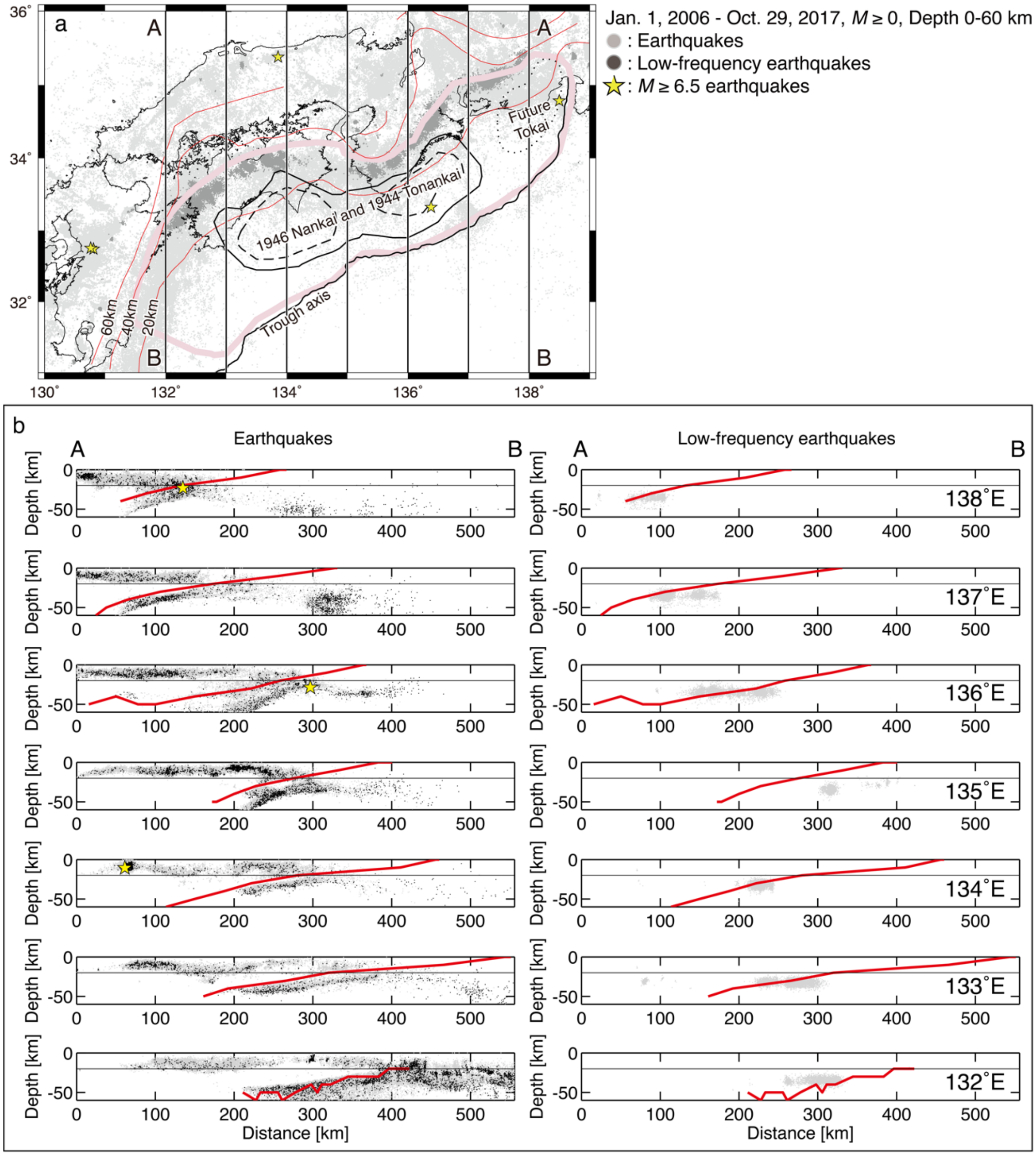

Supplementary Figure 1. Seismicity in the slab along the Nankai Trough. **a**, Seismicity map created by using earthquake data given at the top right of this figure (also see Data availability). Thick purple line: maximum focal region of a megathrust earthquake presented by the Central Disaster Management Council of the Japanese Government[14]. Dotted line: region supposed to be sources of the anticipated Tokai earthquake[14]. Solid and dashed lines: regions of estimated large slips (> 2 and 4 m, respectively) resulting from the 1946 Nankai and 1944 Tonankai earthquakes[12]. Thin red lines: contours of the

subducting slab[40-43] (also see Data availability). Seismicity in the zones with a width of 100 km along seven longitudinal lines from 36°N (A) to 31.0°N (B) was used to create cross-section view in **b**. The map displaying all datasets was produced by using GMT software[53]. **b**, Cross-sectional view of the hypocentral distribution of earthquakes (left column) and low-frequency earthquakes (right column) in each north-south zone with a width of 100 km. In the left column, grey and black dots show earthquakes with $2 > M \geq 0$ and $M \geq 2$, respectively. Red curve represents plate interface[40-43]; Horizontal line is drawn at a depth of 20 km. Also see Methods.

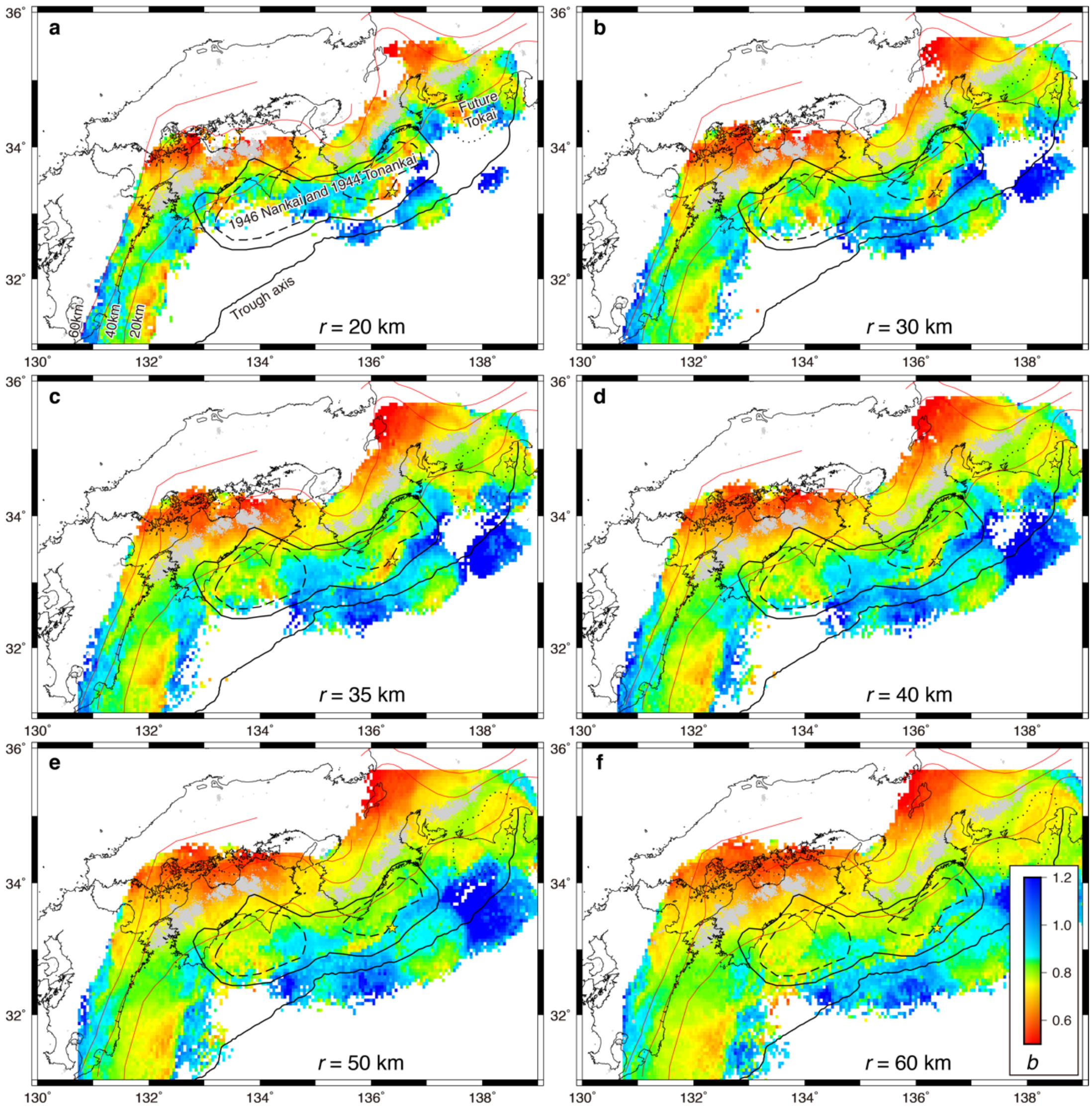

Supplementary Figure 2. *b*-value maps for different sampling radii. The software package ZMAP[47] was used to create these maps on an interactively defined radius *r*. We varied the sampling radius: (**a**) $r = 20$ km, (**b**) $r = 30$ km, (**c**) $r = 35$ km (**d**) $r = 40$ km, (**e**) $r = 50$ km, and (**f**) $r = 60$ km. **c** is the same as Fig. 1. Earthquake data used to create maps in **a**, **b**, **d**, **e**, and **f** are the same as those in **c**. Dotted black line: region supposed to be sources of the anticipated Tokai earthquake[14]. Solid and dashed black lines: regions of estimated large slips (> 2 and 4 m, respectively) resulting from the 1946 Nankai and 1944 Tonankai earthquakes[12]. Thin red lines: contours of the subducting slab[40-43] (also see Data availability). Grey dots: low-frequency earthquakes. Stars: *M*6.5 earthquakes. Also see Methods.

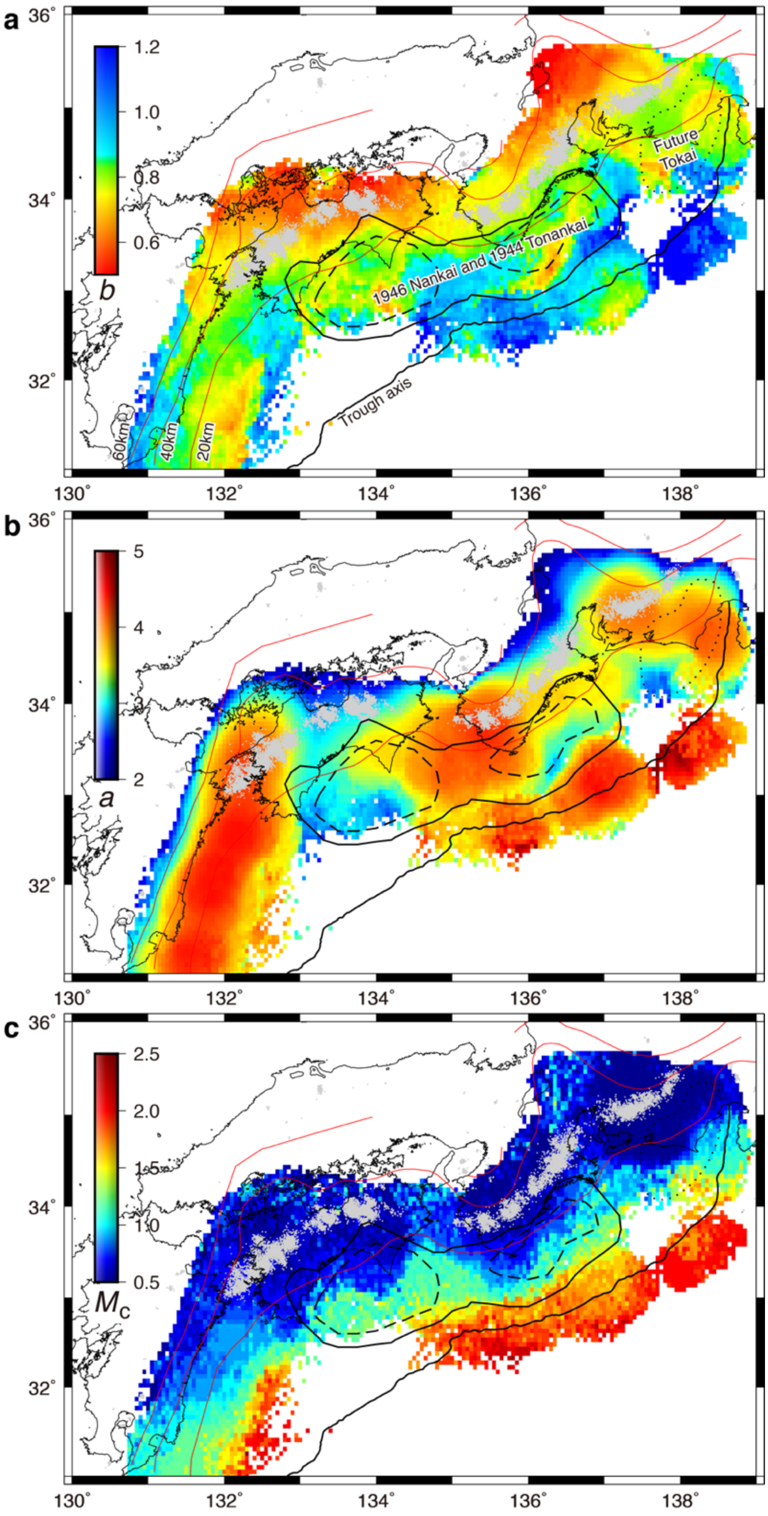

Supplementary Figure 3. Maps of b-value in **a**, a-value in **b**, and M_c in **c**. The b-value map in **a** is the same as that in Fig. 1. Not only b-value but also a-value and M_c are simultaneously calculated by using the EMR method[46]. The software package ZMAP[47]

that includes EMR as one of the standard tools was used to create the maps in **a**-**c**. Dotted black line, solid black line, dashed black lines, thin red lines, and grey dots are the same as those in Supplementary Fig. 2. Also see Methods.

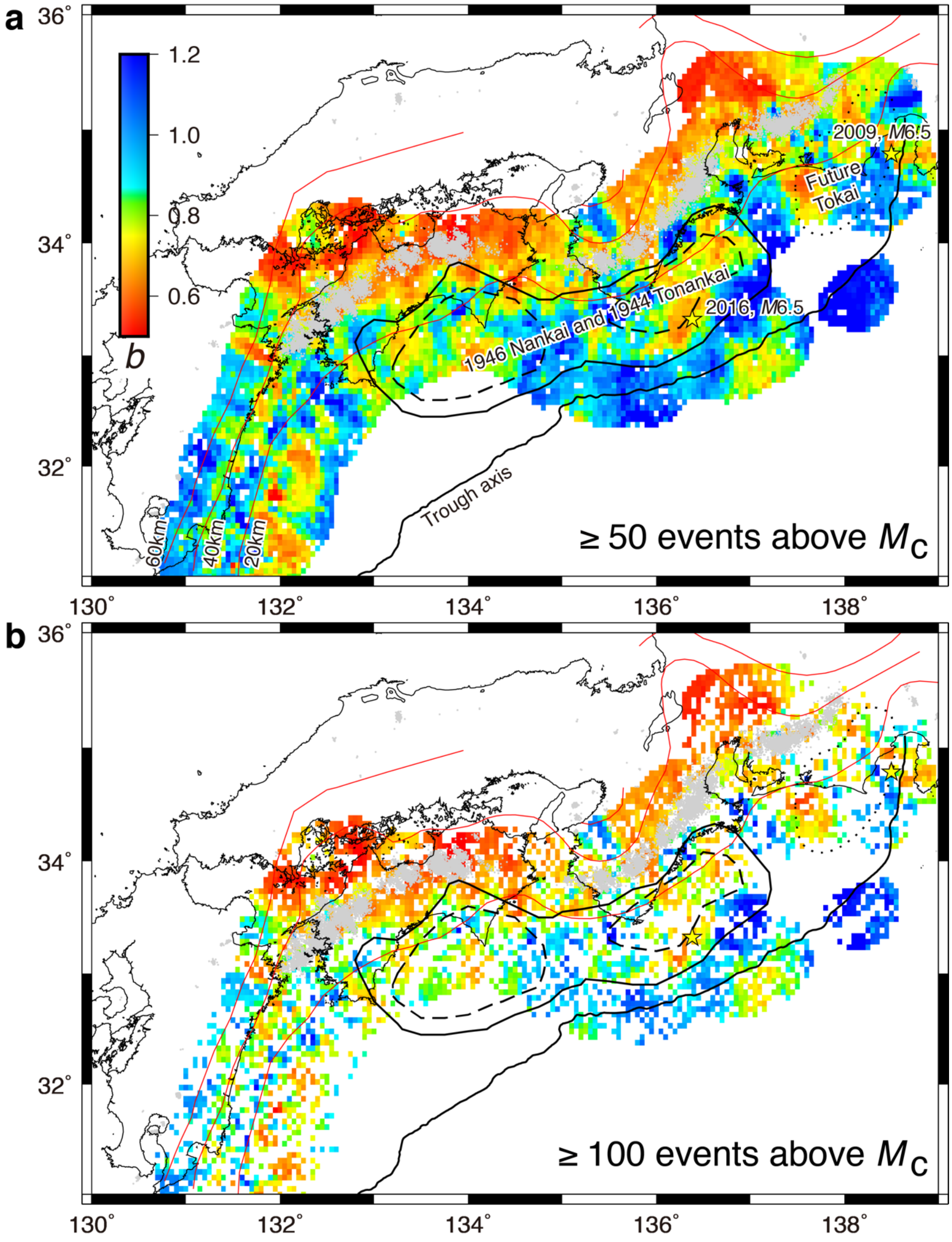

Supplementary Figure 4. Maps of *b* values for different sampling number of earthquakes. **a,** A *b*-value map obtained by using the closest 200 earthquakes to each grid node (grid spacing of 0.05 x 0.05 degrees) with the sampling radius of $r \leq 35$ km, for which at least $N_{min} = 50$ events yield a good fit to the GR law. Earthquake data used

for this map are the same as those for Fig. 1. The software package ZMAP[47] was first used to compute b values for all nodes. Then, the nodes, for which $r > 35$ km was required to sample the closest 200 earthquakes, were removed from the mapping. **b,** Same as **a** for $N_{\mathrm{min}} = 100$. Dotted black line, solid black line, dashed black lines, thin red lines, and grey dots are the same as those in Supplementary Fig. 2. Also see Methods.

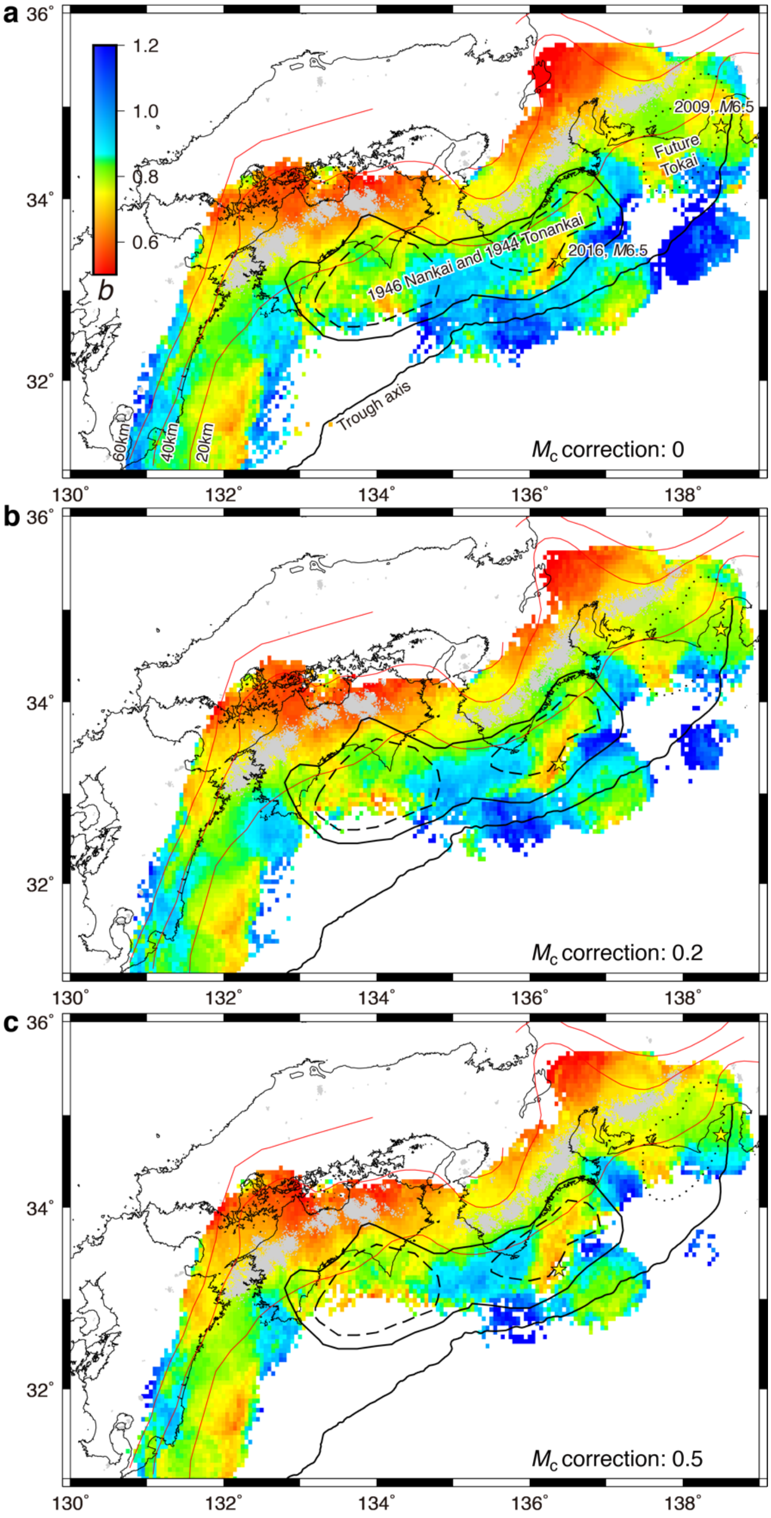

Supplementary Figure 5. b-value map for different values of M_c correction. **a**, Same as Fig. 1 (no M_c correction). **b** and **c**, Maps when M_c is increased by 0.2 and 0.5, respectively. Using the software package ZMAP[47], we interactively increased every

local M_c by the prescribed value, 0.2 in **b** and 0.5 in **c**. Then, b values were computed for every node as performed in creating the map in **a**. Earthquake data used to create these maps in **b** and **c** are the same as those to create the map in **a**. Dotted black line, solid black line, dashed black lines, thin red lines, and grey dots are the same as those in Supplementary Fig. 2. Also see Methods.

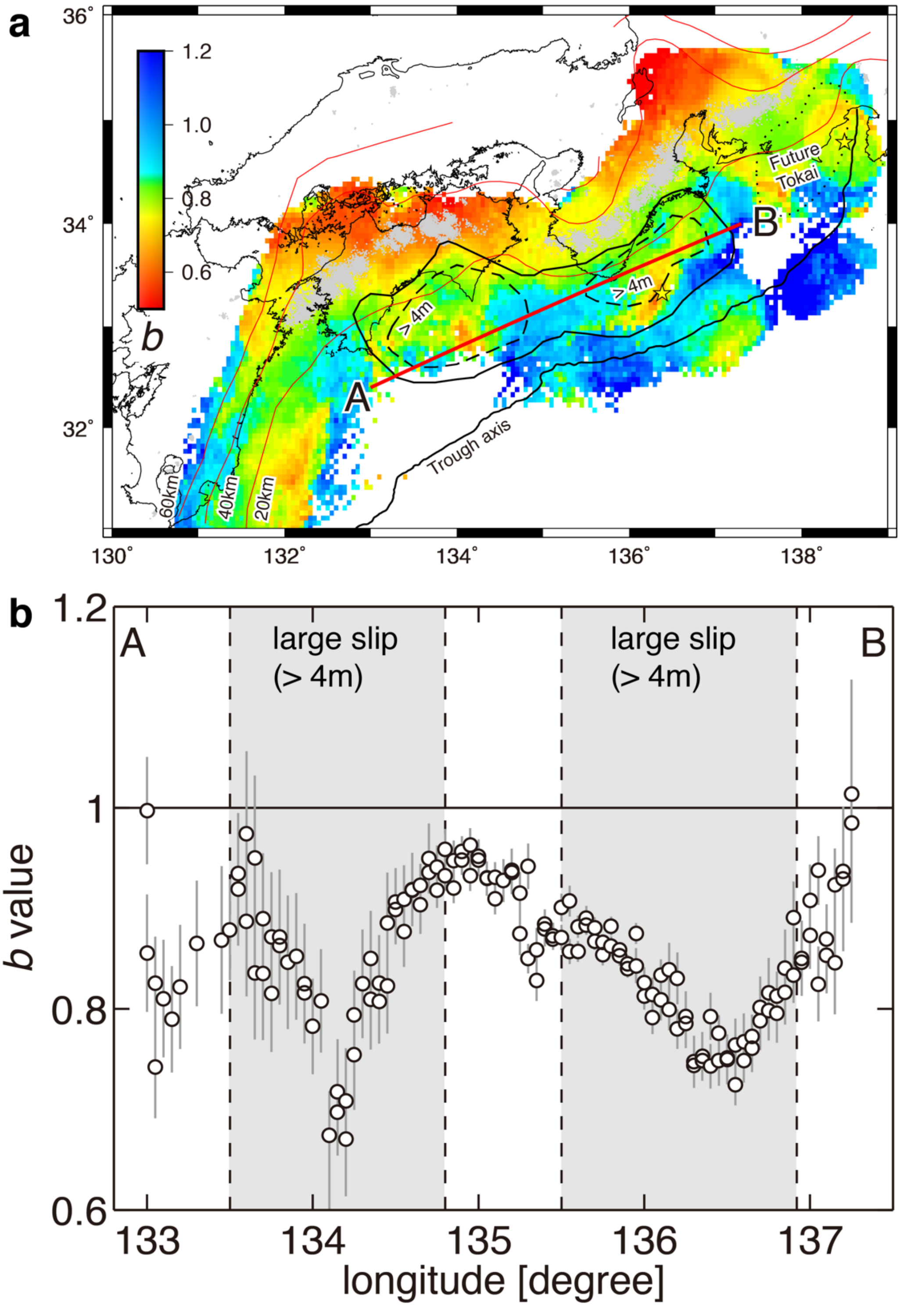

Supplementary Figure 6. Change in the *b* value along the line that passes through the focal regions of the 1946 Nankai and 1944 Tonankai earthquakes. **a,** Same as Fig. 1, but we overlaid the thick red line from A to B, almost parallel to the Trough axis, which

passes through estimated large-slip areas (> 2 m and > 4 m), denoted by solid and dashed lines, respectively, of the 1946 Nankai and 1944 Tonankai earthquakes[12]. Dotted black line, thin red lines, and grey dots are the same as in Supplementary Fig. 2. Stars: *M*6.5 earthquakes. The map displaying all datasets was produced by using GMT software[53]. **b**, Change in the *b* value along the thick red line from A to B in **a**. Grey zones in **b** correspond to large slip areas (> 4 m) of the two earthquakes. The error bars represent one standard deviation of a bootstrap sampling distribution. Also see Methods.

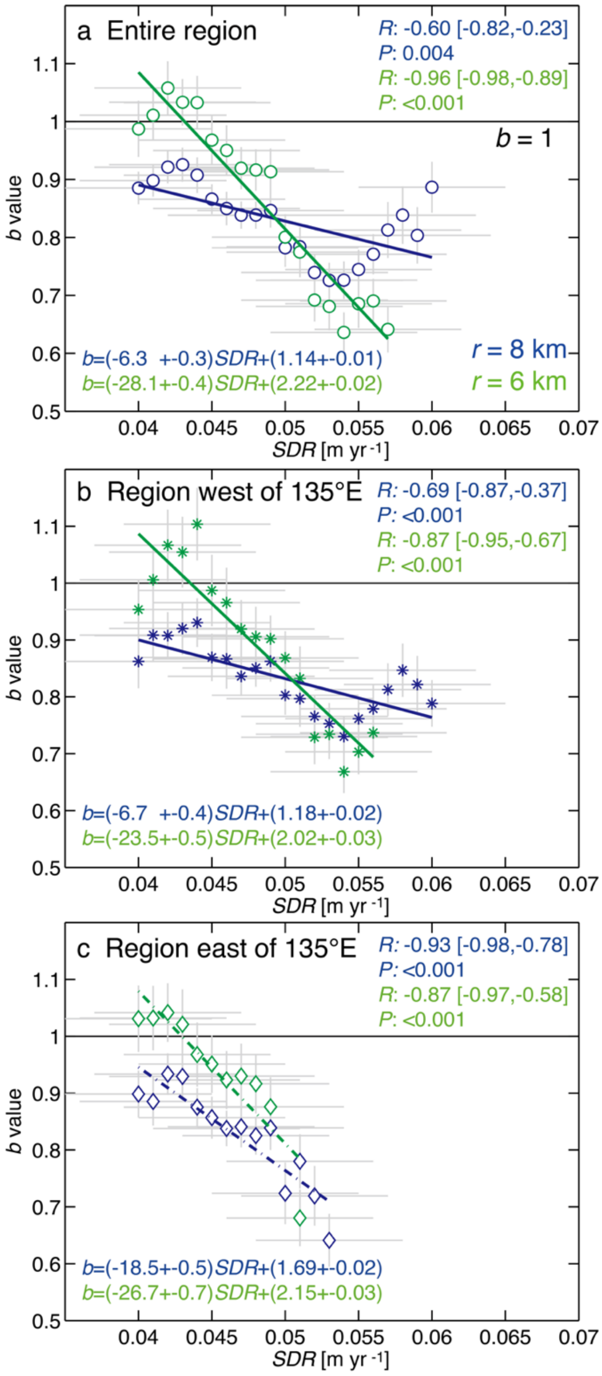

Supplementary Figure 7. *b* versus SDR plot for a window 0.01 m year^{-1}. **a-c**, Same as Figs. **2a-c**, respectively, except for using a narrower window width (horizontal bars in grey). Vertical bars in grey: bootstrapping errors displaying one standard deviation of bootstrap uncertainty. *R*: correlation coefficient; *P*: the *P* value of a significant test. Also see Methods.

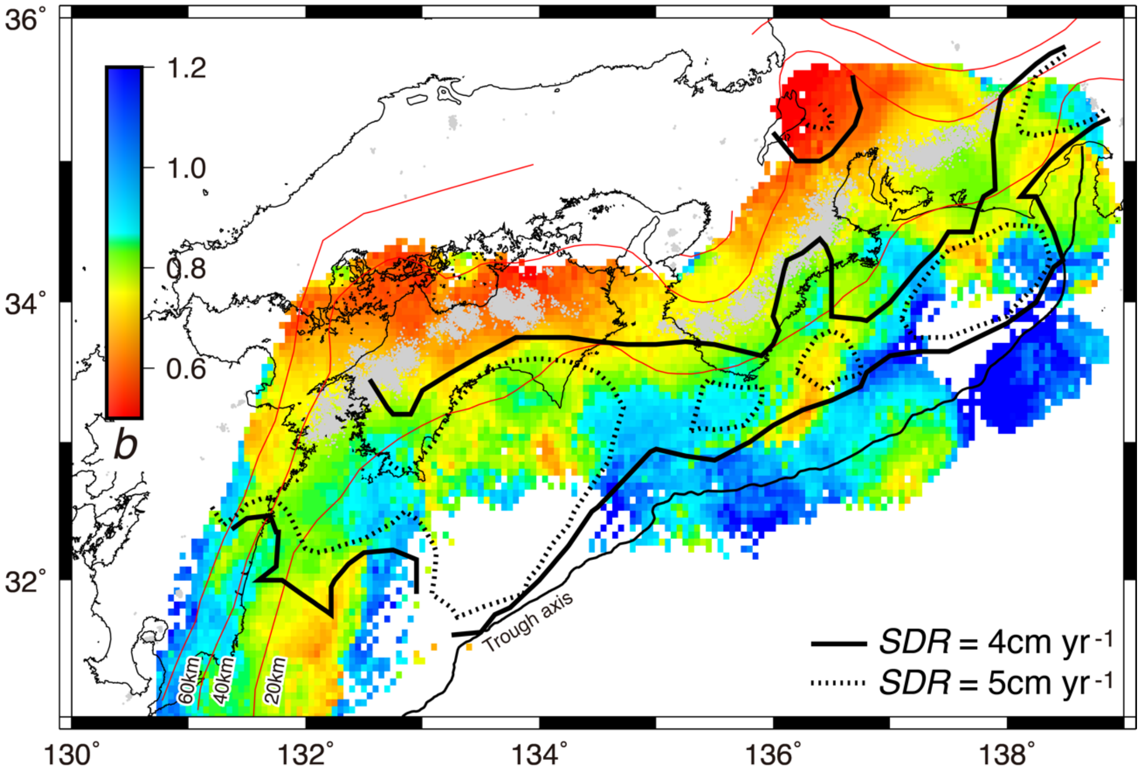

Supplementary Figure 8. *b*-value map with contour lines of SDR. This *b*-value map is the same as that in Fig. 1. On this map, we overlaid contour lines of *SDR* of 4 cm year^{-1} and 5 cm year^{-1} that were shown in Fig. 2 of Yokota et al.[23] (also see Data availability). Thin red lines and grey dots are the same as those in Supplementary Fig. 2. The map displaying all datasets was produced by using GMT software[53]. Also see Methods.

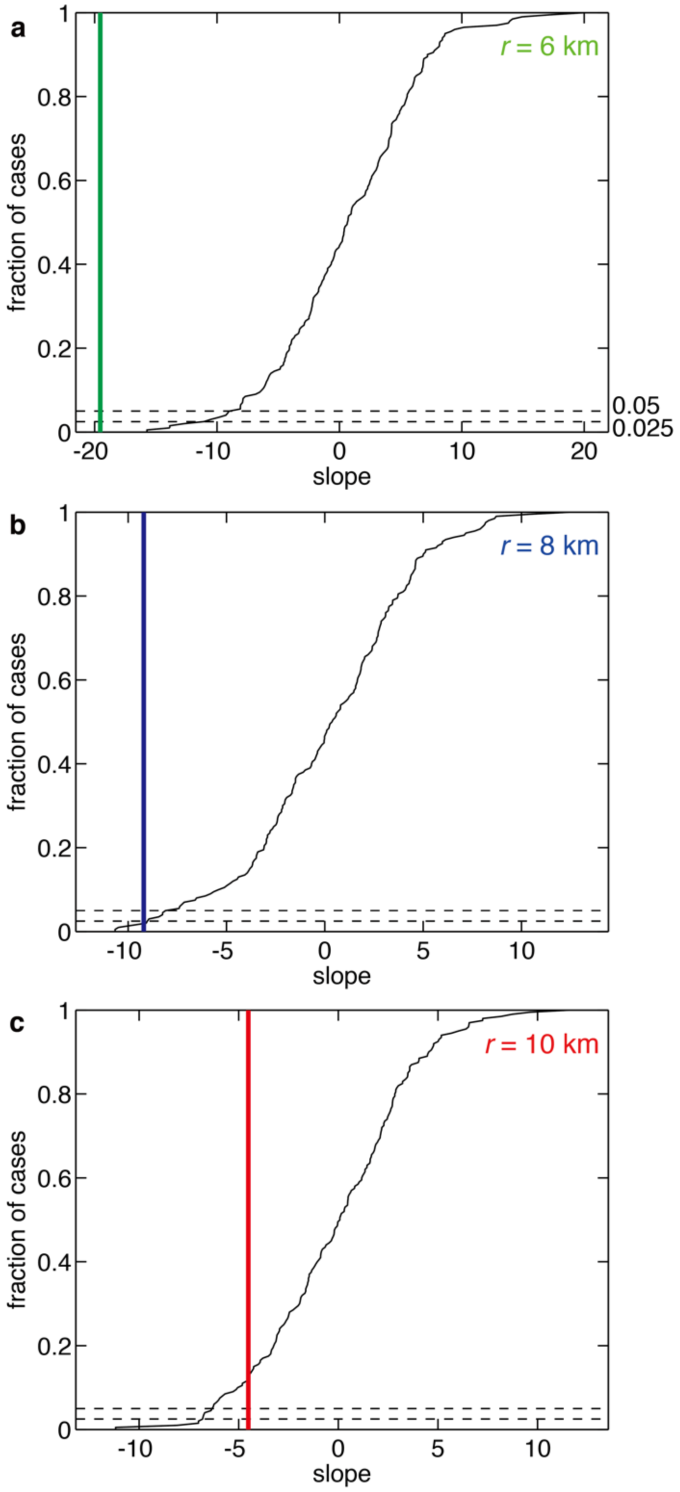

Supplementary Figure 9. Significant test for the inverse relationship between the *b* value and SDR. **a,** The black curve indicates cumulative distribution of slopes of the least-square regression lines based on randomized catalogs for *r* = 6 km. Vertical line:

slope of the least-square regression line in Figure 2a. Horizontal dashed lines: 2.5% and 5% of the cumulative distribution. **b** and **c,** Same as **a** for r = 8 and 10 km, respectively. The vertical line in **c** shows the slope of the least-square regression line for the observation when the radius r = 10 km was taken. Also see Methods.

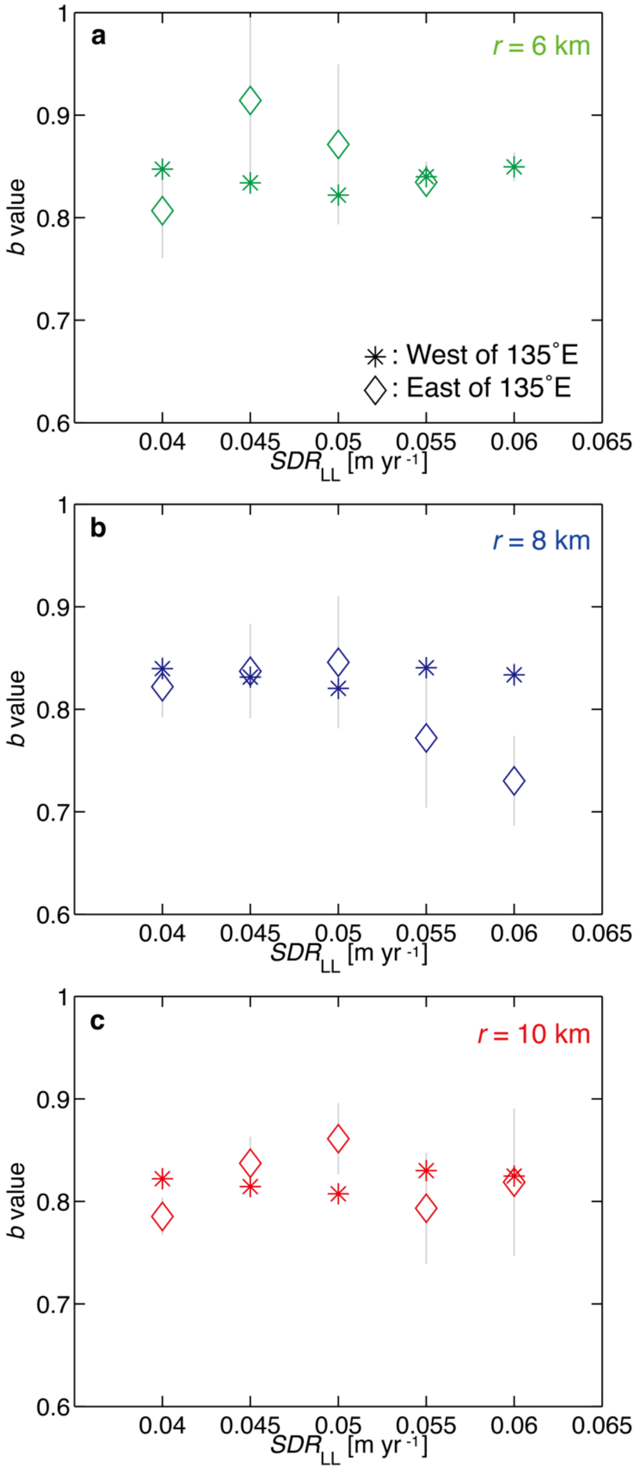

Supplementary Figure 10. b versus SDR_{LL} plot for different r. **a,** $r = 6$ km, **b,** $r = 8$ km, and **c,** $r = 10$ km. **b** is the same as Fig. 3, but the results for $SDR_{LL} = 0.04$ and 0.045 m year^{-1} are added. Vertical bars in grey: bootstrapping errors displaying one standard

deviation of the sample of *b* values that are computed based on the bootstrapped data. For SDR_{LL} = 0.05, 0.055, and 0.06 m year^{-1} in **b**, we got a slightly different result from that shown in Fig. 3, because we ran a simulation using a different seed in bootstrapping calculation from that used for Fig. 3.
